# Supplementary material for: Multi‐dataset identification of innovative feature genes and molecular mechanisms in keratoconus
Source: J Cell Mol Med. 2024 Sep 19;28(18):e70079. doi: 10.1111/jcmm.70079 (PMC11412914; doi:10.1111/jcmm.70079)
Supplement: Supplementary file 2 — Material S2. [file JCMM-28-e70079-s002.zip › 7.GSEA analysis/3.DBNDD1/KEGG_HYPERTROPHIC_CARDIOMYOPATHY_HCM.html]

Details for gene set KEGG\_HYPERTROPHIC\_CARDIOMYOPATHY\_HCM[GSEA]

|  || Dataset | DBNDD1.group.cls#h\_versus\_l |
| Phenotype | group.cls#h\_versus\_l |
| Upregulated in class | l |
| GeneSet | KEGG\_HYPERTROPHIC\_CARDIOMYOPATHY\_HCM |
| Enrichment Score (ES) | -0.39374658 |
| Normalized Enrichment Score (NES) | -1.340889 |
| Nominal p-value | 0.08730159 |
| FDR q-value | 0.8289331 |
| FWER p-Value | 0.944 |
Table: GSEA Results Summary

  

Fig 1: Enrichment plot: KEGG\_HYPERTROPHIC\_CARDIOMYOPATHY\_HCM      
 Profile of the Running ES Score & Positions of GeneSet Members on the Rank Ordered List

  

| SYMBOL | TITLE | RANK IN GENE LIST | RANK METRIC SCORE | RUNNING ES | CORE ENRICHMENT || 1 | TNNT2 | na | 514 | 0.448 | 0.0223 | No |
| 2 | CACNB1 | na | 587 | 0.431 | 0.0559 | No |
| 3 | TNNI3 | na | 657 | 0.420 | 0.0887 | No |
| 4 | CACNA1F | na | 1766 | 0.316 | 0.0828 | No |
| 5 | ACTC1 | na | 2612 | 0.267 | 0.0806 | No |
| 6 | CACNG5 | na | 2873 | 0.257 | 0.0943 | No |
| 7 | CACNG7 | na | 4144 | 0.209 | 0.0750 | No |
| 8 | SGCD | na | 4837 | 0.185 | 0.0703 | No |
| 9 | TPM2 | na | 5034 | 0.180 | 0.0795 | No |
| 10 | DAG1 | na | 5307 | 0.170 | 0.0858 | No |
| 11 | ITGA4 | na | 7459 | 0.114 | 0.0331 | No |
| 12 | TNNC1 | na | 7636 | 0.111 | 0.0373 | No |
| 13 | EMD | na | 7789 | 0.108 | 0.0418 | No |
| 14 | TTN | na | 7964 | 0.104 | 0.0454 | No |
| 15 | CACNG1 | na | 8316 | 0.098 | 0.0434 | No |
| 16 | LAMA2 | na | 9618 | 0.077 | 0.0122 | No |
| 17 | ITGB7 | na | 9817 | 0.074 | 0.0126 | No |
| 18 | CACNA2D3 | na | 10208 | 0.068 | 0.0070 | No |
| 19 | IGF1 | na | 10555 | 0.064 | 0.0023 | No |
| 20 | ATP2A2 | na | 11124 | 0.057 | -0.0094 | No |
| 21 | CACNA1D | na | 13311 | 0.036 | -0.0695 | No |
| 22 | DES | na | 13515 | 0.034 | -0.0726 | No |
| 23 | TGFB3 | na | 13624 | 0.033 | -0.0729 | No |
| 24 | CACNB2 | na | 14012 | 0.030 | -0.0816 | No |
| 25 | MYBPC3 | na | 14239 | 0.028 | -0.0858 | No |
| 26 | TPM4 | na | 14283 | 0.028 | -0.0848 | No |
| 27 | ACTB | na | 14636 | 0.025 | -0.0928 | No |
| 28 | PRKAG1 | na | 14780 | 0.024 | -0.0949 | No |
| 29 | CACNG8 | na | 15428 | 0.020 | -0.1120 | No |
| 30 | DMD | na | 15967 | 0.016 | -0.1262 | No |
| 31 | PRKAG3 | na | 16144 | 0.015 | -0.1300 | No |
| 32 | ITGA2B | na | 16436 | 0.013 | -0.1373 | No |
| 33 | PRKAB1 | na | 16608 | 0.012 | -0.1413 | No |
| 34 | PRKAG2 | na | 17777 | 0.004 | -0.1746 | No |
| 35 | TPM1 | na | 17928 | 0.003 | -0.1787 | No |
| 36 | ACE | na | 18072 | 0.002 | -0.1827 | No |
| 37 | ACTG1 | na | 18138 | 0.002 | -0.1844 | No |
| 38 | TGFB2 | na | 19303 | -0.007 | -0.2174 | No |
| 39 | LMNA | na | 20345 | -0.016 | -0.2461 | No |
| 40 | PRKAA1 | na | 20353 | -0.016 | -0.2450 | No |
| 41 | ITGB4 | na | 20511 | -0.017 | -0.2481 | No |
| 42 | ITGA9 | na | 21410 | -0.026 | -0.2719 | No |
| 43 | ITGB5 | na | 21935 | -0.031 | -0.2844 | No |
| 44 | PRKAB2 | na | 22826 | -0.041 | -0.3067 | No |
| 45 | TPM3 | na | 23026 | -0.043 | -0.3088 | No |
| 46 | CACNA1C | na | 23124 | -0.045 | -0.3079 | No |
| 47 | MYH7 | na | 23841 | -0.054 | -0.3241 | No |
| 48 | ITGA3 | na | 24015 | -0.056 | -0.3244 | No |
| 49 | IL6 | na | 24766 | -0.067 | -0.3405 | No |
| 50 | PRKAA2 | na | 24976 | -0.070 | -0.3407 | No |
| 51 | MYL2 | na | 25552 | -0.079 | -0.3507 | No |
| 52 | MYL3 | na | 25616 | -0.081 | -0.3459 | No |
| 53 | ITGAV | na | 25782 | -0.083 | -0.3437 | No |
| 54 | ITGA11 | na | 26521 | -0.098 | -0.3569 | No |
| 55 | ITGA2 | na | 26752 | -0.102 | -0.3551 | No |
| 56 | TNF | na | 27164 | -0.111 | -0.3577 | No |
| 57 | CACNG4 | na | 28414 | -0.141 | -0.3821 | Yes |
| 58 | ITGA6 | na | 28732 | -0.150 | -0.3788 | Yes |
| 59 | ITGA7 | na | 29075 | -0.159 | -0.3755 | Yes |
| 60 | ITGB1 | na | 29102 | -0.160 | -0.3630 | Yes |
| 61 | SGCA | na | 29145 | -0.161 | -0.3509 | Yes |
| 62 | SGCB | na | 29234 | -0.164 | -0.3399 | Yes |
| 63 | CACNB3 | na | 29250 | -0.164 | -0.3268 | Yes |
| 64 | CACNA2D4 | na | 29251 | -0.164 | -0.3131 | Yes |
| 65 | ITGB3 | na | 29286 | -0.165 | -0.3004 | Yes |
| 66 | MYH6 | na | 29406 | -0.169 | -0.2899 | Yes |
| 67 | ITGB6 | na | 29433 | -0.170 | -0.2765 | Yes |
| 68 | SGCG | na | 29686 | -0.179 | -0.2690 | Yes |
| 69 | ITGB8 | na | 30390 | -0.200 | -0.2727 | Yes |
| 70 | TGFB1 | na | 30914 | -0.218 | -0.2698 | Yes |
| 71 | ITGA1 | na | 31047 | -0.222 | -0.2552 | Yes |
| 72 | CACNG6 | na | 31154 | -0.226 | -0.2395 | Yes |
| 73 | SLC8A1 | na | 32046 | -0.266 | -0.2432 | Yes |
| 74 | RYR2 | na | 32172 | -0.274 | -0.2241 | Yes |
| 75 | ITGA10 | na | 32340 | -0.284 | -0.2053 | Yes |
| 76 | CACNA2D2 | na | 32648 | -0.304 | -0.1890 | Yes |
| 77 | CACNG2 | na | 33176 | -0.343 | -0.1759 | Yes |
| 78 | CACNA2D1 | na | 33832 | -0.408 | -0.1610 | Yes |
| 79 | CACNB4 | na | 33927 | -0.420 | -0.1290 | Yes |
| 80 | ITGA5 | na | 34082 | -0.440 | -0.0970 | Yes |
| 81 | ITGA8 | na | 34117 | -0.445 | -0.0611 | Yes |
| 82 | CACNA1S | na | 34256 | -0.474 | -0.0258 | Yes |
| 83 | CACNG3 | na | 34262 | -0.475 | 0.0135 | Yes |
Table: GSEA details [plain text format]

  

Fig 2: KEGG\_HYPERTROPHIC\_CARDIOMYOPATHY\_HCM      
 Blue-Pink O' Gram in the Space of the Analyzed GeneSet

  

Fig 3: KEGG\_HYPERTROPHIC\_CARDIOMYOPATHY\_HCM: Random ES distribution      
 Gene set null distribution of ES for **KEGG\_HYPERTROPHIC\_CARDIOMYOPATHY\_HCM**
